# Clinical Experience of Bronchoscopy-Guided Radiofrequency Ablation for Peripheral-Type Lung Cancer

**DOI:** 10.1155/2013/515160

**Published:** 2013-09-11

**Authors:** Tomonobu Koizumi, Takashi Kobayashi, Tsuyoshi Tanabe, Kenji Tsushima, Masanori Yasuo

**Affiliations:** ^1^Department of Comprehensive Cancer Therapy, Shinshu University School of Medicine, 3-1-1 Asahi, Matsumoto 390-8621, Japan; ^2^First Department of Internal Medicine, Shinshu University School of Medicine, 3-1-1 Asahi, Matsumoto 390-8621, Japan

## Abstract

We have developed a new internal cooled electrode for radiofrequency ablation (RFA) (Japan Application no. 2006-88228) suitable for forceps channel bronchoscopy. Here, we present our clinical experience with bronchoscopy-guided RFA under computed tomography (CT) monitoring for patients with peripheral-type non-small-cell lung cancer (NSCLC). Bronchoscopy-guided RFA was performed in two patients (80 and 70 years old) with NSCLC, who had no lymph node involvement and distant metastases (T1N0M0), but not indicated for surgery because of other complications, such as advanced age, poor pulmonary function, and refusal of thoracic surgery. The locations of the tumors were right S2 and left S3, respectively. Although the tumors showed ground-glass opacity (GGO) with solid components in both cases, radiographic findings changed to reduced mass-like shadow and remained stable for 4 and 3.5 years after bronchoscopy-guided RFA. As the former case developed progressive disease on chest CT, bronchoscopy-guided RFA was repeated in the same lesion, resulting in no change for the subsequent 1 year. There were no adverse reactions during the procedures. Thus, bronchoscopy-guided RFA is a safe and feasible procedure that represents a potentially useful therapeutic tool in local control in medically inoperable patients with stage I NSCLC.

## 1. Introduction

Surgical resection is the standard treatment for patients with stage I non-small-cell lung cancer (NSCLC). However, thoracic surgery is often not feasible in high-risk patients. Stereotactic body radiotherapy (SBRT) and percutaneous radiofrequency ablation (RFA) therapy are options for medically inoperable patients with early stage NSCLC. Miao et al. suggested that cooled RFA is an effective alternative to lobectomy in certain patients for minimally invasive treatment of lung cancer [1]. In general, standard RFA has been widely used as percutaneous imaging-guided therapy [2, 3]. Complications of this method include pain, pneumothorax, hemothorax, and pleural effusion [2–4]. Tsushima et al. reported fiberoptic bronchoscopy guidance under real-time computed tomography (CT) fluoroscopy for the diagnosis of peripheral lung lesions [5]. A critical aspect is that this procedure may reduce the above complications if we can apply RFA using real-time chest CT images under fiberoptic bronchoscopy. We have developed a new internal cooled electrode suitable for forceps channel bronchoscopy (Japan Application no. 2006-88228). Using this technique, we previously reported that internal bronchoscopy-guided cooled RFA was a safe, effective, and feasible procedure without major complications in the normal sheep lung [6] and in a preclinical study [7]. Subsequently, we advanced our procedures in clinical practice. Here, we introduce our experience with bronchoscopy-guided RFA therapy for two medically inoperable patients with early stage and peripheral type NSCLC.

## 2. Methods

**Figure 1:**
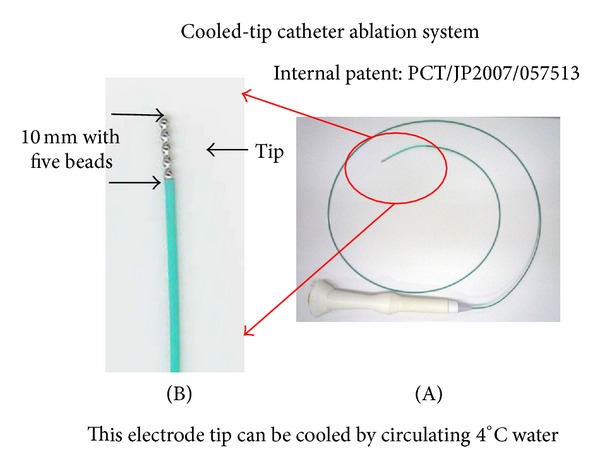
(A) This model is an internal cooled radiofrequency ablation electrode. (B) The top of the electrode. Only a 10 mm electrode produced power output, and the tip temperature and impedance were measured.

**Figure 2:**
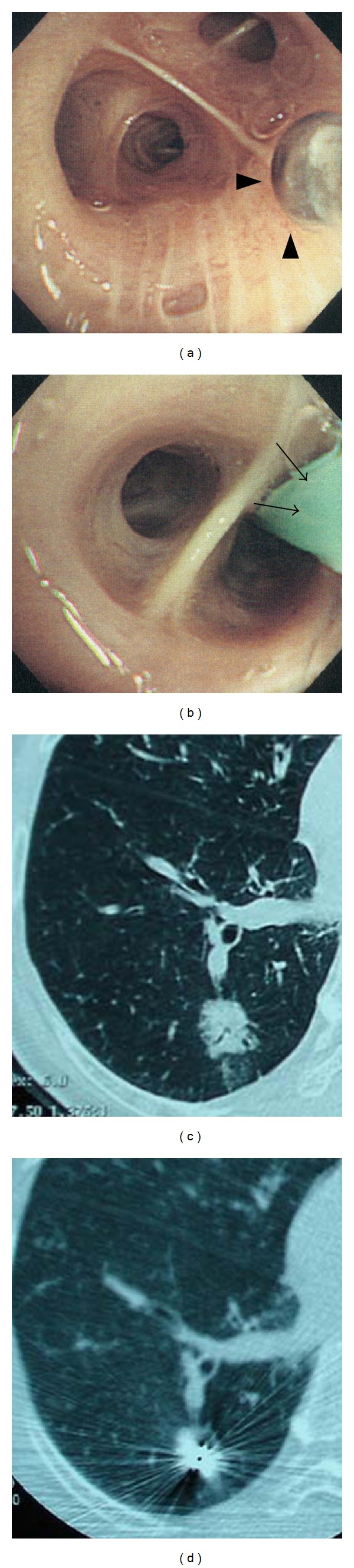
((a), (b)) Flexible fiberoptic bronchoscope (Olympus; Tokyo, Japan) BF 260 (outer diameter: 4.9 mm, forceps channel: 2.0 mm). 5 Fr cooled catheter tip was inserted under fluoroscopic guidance. (a) The tip of the electrode (arrowheads). (b) Shaft of the electrode (arrows). ((c), (d)) Low-dose CT guidance was performed to correlate the location of the catheter tip in the lung tumor. (c) Peripheral lung tumor (ground-glass opacity) (arrow) on low-dose CT image. (d) Halation around tumor on CT image reflected the catheter tip.

Our cooled-tip catheter ablation electrode is shown in Figure 1. The tip length is 10 mm with 5 beads. We inserted this catheter into the bronchoscopy channel and approached the tumor. The electrode was attached to a monopolar radiofrequency generator (Shinshu University, Nagano, Japan) capable of producing 50 W as the maximum output. Tissue impedance was monitored continuously by a generator, and an impedance-controlled radiofrequency algorithm was used. During the RFA procedure, a thermometer embedded within the electrode tip continuously measured the temperature, and its upper limit was set at 70°C. Grounding was achieved by attaching one or two standard steel mesh dispersive electrodes to the lumbar region in patients. A peristaltic pump (Shinshu University, Nagano, Japan) was used to infuse cold water (4°C) into the internal lumen of the catheter electrode at a constant rate (50 mL/min). The cooled water was used to avoid the pop phenomenon. When the desired power output could not be applied because of elevation of impedance due to tissue boiling, the generator automatically switched the electrode off. Current pulsing was also performed manually to avoid charring local tissue caused by the rapid increase in impedance, which limited further heat diffusion. The practice of our CT-guided bronchoscopy-guided cooled RFA is shown in Figure 2. We advanced the catheter into the bronchus affected by the malignant tumor and fixed the position with CT images. Then, we performed RFA under CT guidance. A CT fluoroscopic image was obtained to correlate the location of the electrode tip in peripheral lung lesions with approximately 1 cm margin from the pleura to prevent pneumothorax. This study was performed with the approval of our institutional human studies committee, and written informed consent was obtained from each patient. RFA output power in the generator was fixed to 30 W and ablation interval was 50 s.

## 3. Case Presentation

### 3.1. Case 1

**Figure 3:**
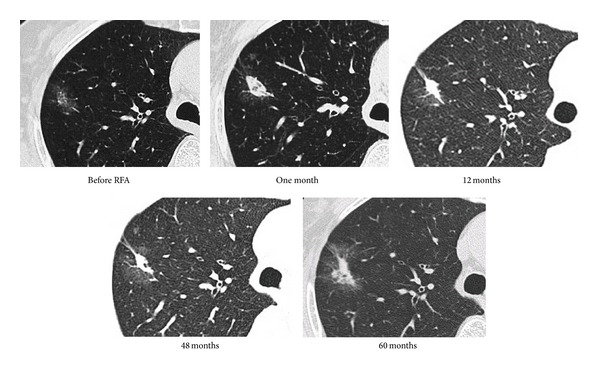
Serial CT findings before and after initial bronchoscopy-guided cooled radiofrequency ablation (RFA) in case 1.

**Figure 4:**
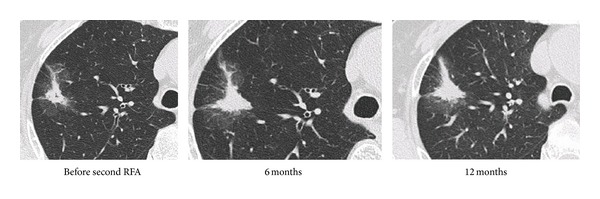
Serial CT findings before and after second bronchoscopy-guided cooled radiofrequency ablation (RFA) in case 1.

An 80-year-old woman was initially admitted to hospital because of abnormal findings on chest radiography screening. She had a history of left lower lobectomy for primary lung adenocarcinoma (T1N0M0) and cerebral infarction six and three years ago, respectively. She had slight right hemiparesis, but was asymptomatic. Chest radiography and CT revealed a 20 mm nodule in right S2 without any hilar or mediastinal lymphadenopathy. Bronchoscopic examination revealed epidermal growth factor receptor wild-type adenocarcinoma cells from the nodule. Bronchoscopy-guided RFA was performed because of her poor pulmonary function and advanced age. The time courses of radiographic findings on chest CT before and after RFA are shown in Figure 3. The radiographic findings changed to be a mass-like shadow after RFA and remained stable in size for 4 years. However, the mass increased in size and the bronchial lumen in the mass became narrow, which were considered to be indicators of progressive disease (60 months after initial RFA). Bronchoscopy-guided RFA was then repeated, and the mass shadow remained stable for 12 months (Figure 4).

### 3.2. Case 2

**Figure 5:**
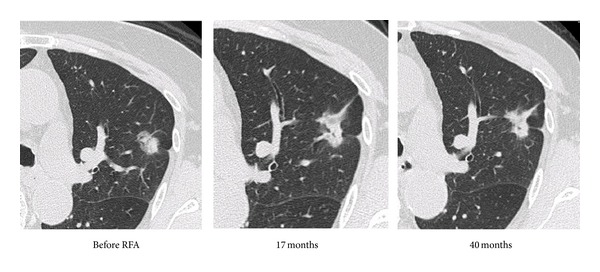
Serial CT findings before and after bronchoscopy-guided cooled radiofrequency ablation (RFA) in case 2.

A 70-year-old woman, who had undergone radical hysterectomy for cervical cancer in our hospital, was referred to our respiratory center because of abnormal findings on chest CT. The abnormal findings were noted by systemic review before surgery. Bronchofiberscopy was performed, and a diagnosis of primary adenocarcinoma in the lung was made. There were no hilar or mediastinal lymph node metastases. She was treated with internal RFA as she refused thoracic surgery. Serial chest CT findings before and after RFA were shown in Figure 5. Tumor shadow, showing ground-glass opacity (GGO) with solid components, was observed in left S3 and changed to a mass-like shadow after internal RFA. The mass remained stable for 40 months.

## 4. Discussion

Percutaneous guided-RFA has clinical applications in lung cancer, and good results have been reported [1–4, 8]. As the electrode is placed percutaneously directly into the tumor under cross-sectional imaging guidance, such as chest CT, complications such as pneumothorax occur at relatively high rates. The frequency of pneumothorax or hemothorax with use of percutaneous direct electrode placement was 47% in a rabbit model [9]. Clinical complications occurring in percutaneously directly RFA were 16%–35% [3, 4]. However, it is possible to avoid these complications using fiberoptic bronchoscopy guidance; this is the greatest advantage of our internal cooled electrode. Our fiberscopic bronchoscopy-guided cooled RFA is safe and technically feasible as we confirm placement of the tip of the electrode by CT or X-ray imaging guidance. To our knowledge, this is the first report of bronchoscopyc-guided cooled RFA as a potential therapeutic tool.

 This internal cooled RFA is constructed as a thin catheter (diameter: 1.67 mm) because it passes through the bronchoscopy channel. The necrotic size obtained by this electrode is a critical consideration for this method. To achieve higher power output from the electrode and sufficient coagulation necrosis, it is necessary to increase the power output and reduce impedance around the electrode tip. Using the standard noncooled electrode, the temperature around the tip rose rapidly and the pop phenomenon occurred in the lung tissues. This phenomenon means that coagulated necrotic tissue is formed around the electrode tip and tissue impedance increases rapidly. To avoid this phenomenon, the electrode tip should be cooled using water. Based on these results of using cooled RFA for cardiac conduction disease [10, 11], it is predicted that the lesion occurs in lung tissue away from the cooled RFA. The cooled RFA enables greater power output for a longer time compared with the standard noncooled RFA, resulting in larger area of coagulation necrosis. The electrode tip is cooled by circulating water in the electrode catheter. As a result, the tissue around the electrode tip does not reach an excessively high temperature. Based on this theory, the cooled RFA can reach deeper and wider areas of ablation using the same power output. 

In clinical situations, we have frequently encountered patients with synchronous multiple lung cancer lesions in both lungs, poor cardiopulmonary status, or coexistent medical comorbidities, which are contraindications for thoracic surgery. Bronchoscopy-guided cooled RFA is an option for such cases. The role of bronchoscopy-guided cooled RFA in lung cancer is not sufficiently clear, and the indications for bronchoscopy-guided cooled RFA are limited, with the technique being suitable only in highly selected patients. However, bronchoscopy-guided RFA achieves only local coagulation necrosis, is likely to minimize injury to lung tissue, and may have a good effect on the patient's condition. The advantages of bronchoscopy-guided cooled RFA include the ability to administer treatment nonsurgically and to provide local control of tumors in medically inoperable patients with early stage NSCLC. 

In conclusion, internal bronchoscopy-guided cooled RFA is a safe, effective, and feasible procedure without major complications. Bronchoscopy-guided internal cooled RFA may be an alternative strategy or a potentially useful therapeutic tool, especially for local control in medically inoperable patients with early stage NSCLC.
